# Synthesis and Crystal Structures of Benzimidazole-2-thione Derivatives by Alkylation Reactions

**DOI:** 10.3390/molecules21010012

**Published:** 2015-12-22

**Authors:** El Sayed H. El Ashry, Yeldez El Kilany, Nariman M. Nahas, Assem Barakat, Nadia Al-Qurashi, Hazem A. Ghabbour, Hoong-Kun Fun

**Affiliations:** 1 Chemistry Department, Faculty of Science, Alexandria University, P. O. Box 426, Alexandria 21321, Egypt; yeldez244@hotmail.com (Y.E.K.); ambarakat@ksu.edu.sa (A.B.); 2 Chemistry Department, Faculty of Applied Science, Umm Al-Qura University, Makkah 21955, Saudi Arabia; narinahas@hotmail.com (N.M.N.); maam10@hotmail.com (N.A.-Q.); 3 Department of Chemistry, College of Science, King Saud University, P. O. Box 2455, Riyadh 11451, Saudi Arabia; 4 Department of Pharmaceutical Chemistry, College of Pharmacy, King Saud University, P. O. Box 2457, Riyadh 11451, Saudi Arabia; ghabbourh@yahoo.com (H.A.G.); hfun.c@ksu.edu.sa (H.-K.F.)

**Keywords:** benzimidazole-thione, thiazino[3,2-*a*]benzimidazole, X-ray

## Abstract

Alkylated, benzylated and bromoalkylated benzimidazole-thione that intramolecularly heterocyclized to 3,4-dihydro-2*H*-[1,3]thiazino[3,2-*a*]benzimidazole were synthesized. The chemical structure of the synthesized product was characterized by Infra Red, ^1^H-NMR, ^13^C-NMR, and Mass spectroscopy. Furthermore, the molecular structures of **8** and **9** were confirmed by X-ray single crystallography in different space groups, *Pbca* and *P*2_1_/*c*, respectively.

## 1. Introduction

Benzimidazole is a biologically important scaffold and it is a useful structural motif for the development of molecules of pharmaceutical or biological interest. Appropriately substituted benzimidazole derivatives have found diverse therapeutic applications. It has earned an important place as a pharmacophore in chemotherapeutic agents of pharmacological activities. The biological significance of benzimidazoles can be correlated with its close relationship with the structure of purines that have vital role in the biological system. Moreover, 5,6-dimethyl-1-(α-d-ribofuranosyl)benzimidazole is an integral part of the structure of Vitamin B12. Different pharmacological effects, including antifungal [1], anthelmintic [2], anti-HIV [3], antihistaminic [4,5,6], antiulcer [7,8], cardiotonic [9], antihypertensive [10,11], and neuroleptic [12], have been reported. The optimization of benzimidazole-based structures has resulted in various drugs that are currently on the market, such as omeprazole (proton pump inhibitor), pimobendan (ionodilator), and mebendazole (anthelmintic).

Having the above aspects in mind, our attention has been attracted to synthesizing benzimidazolethione ring and its *N*-acetyl derivative to study their alkylation, aralkylation and bromoalkylation, which subsequently underwent intramolecular cyclisation to give the fused tricyclic ring 3,4-dihydro-2*H*-[1,3]thiazino[3,2-*a*]benzimidazole. The X-ray crystallographic analysis of the alkylated derivatives was also investigated.

## 2. Results

### 2.1. Chemistry

Diversified methods for the preparation of 1*H*-benzo[*d*]imidazole-2(3*H*)-thione **1** have been reported [13,14,15], which can exhibit tautomerism of the type thione-thiol. Its ^1^H-NMR spectrum showed the presence of 2NH as a singlet at δ 12.20 ppm, which disappeared upon addition of D_2_O. The protons of the phenyl ring showed two sets of signals at δ 7.19 and 7.10 ppm. Thus, it existed in the thione form. Its reported [16] X-ray also agreed with the thione tautomer. The minimized energy structure agreed with that of the X-ray, which has been repeated and found to be similar to that reported earlier.

**Scheme 1:**
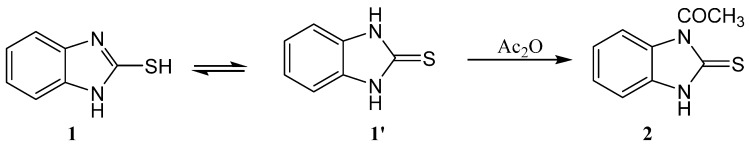
Synthesis of 1-(2-thioxo-2,3-dihydro-1*H*-benzo[*d*]imidazol-1-yl)ethanone **2**.

The reaction of 1*H*-benzo[*d*]imidazole-2(3*H*)-thione **1** with boiling acetic anhydride gave 1-(2-thioxo-2,3-dihydro-1*H*-benzo[*d*]imidazol-1-yl)ethanone **2** (Scheme 1). The product was similar to that prepared in literature [17]. The structure was confirmed from the spectral characteristics. Its IR spectrum showed the presence of broad signal at 1716 (C=O) and 3450 cm^−1^ (NH). Its ^1^H-NMR spectrum showed the presence of CH_3_ as a singlet at δ 1.92 ppm, the signal of NH disappeared because the sample was measured in a solvent mixture including D_2_O. Its ^13^C-NMR spectrum showed the presence of CH_3_ as a singlet at δ 27.1 ppm, the C=O as a singlet at δ 171.1 ppm and C=S as a singlet at δ 168.9 ppm.

Attempted alkylation of **1** and **2** in presence of base gave the same alkylated products, indicating the loss of the acetyl group during the alkylation. Thus, reaction of 1-(2-thioxo-2,3-dihydro-1*H*-benzo[*d*]imidazol-1-yl)ethanone **2** with different bases in acetone as a solvent gave 1*H*-benzo[*d*]imidazole-2(3*H*)-thione **1** whose rate of formation depend on the base and time. Piperidine was found to be the most efficient base for the deacetylation. This was followed by potassium hydroxide, triethylamine and then potassium carbonate. Hydrazine hydrate led to hydrolysis of the acetyl group without further reaction. These results confirmed that our conclusion of losing the acetyl group during the alkylation was due to the base present in the reaction medium.

**Scheme 2:**
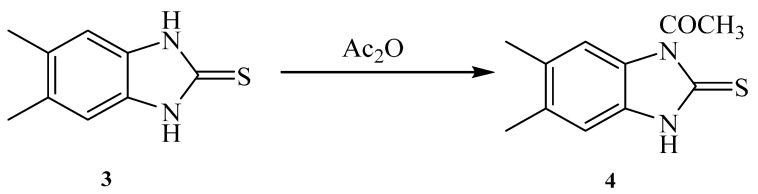
1-(5,6-Dimethyl-2-thioxo-2,3-dihydro-1*H*-benzo[*d*]imidazol-1-yl)ethanone **4**.

Similarly, reaction of 5,6-Dimethyl-1*H*-benzo[*d*]imidazole-2(3*H*)-thione **3** with acetic anhydride gave a product that was identified as 1-(5,6-dimethyl-2-thioxo-2,3-dihydro-1*H*-benzo[*d*]imidazol-1-yl)ethanone **4** (Scheme 2). The chemical structure was deduced from the spectral analysis. IR spectrum showed the presence carbonyl amide at 1685 cm^−1^. Its ^1^H-NMR spectrum showed the presence of two CH_3_ as a singlet at δ 2.22 ppm and NH as a singlet at δ 13.14 ppm. Its ^13^C-NMR spectrum showed the presence of two CH_3_ as two signals at δ 18.6 and 18.9 ppm. The C=S appeared as a signal at 168.4 ppm, and the C=O as a signal at 171.1 ppm.

Reaction of 1-(5,6-dimethyl-2-thioxo-2,3-dihydro-1*H*-benzo[*d*]imidazol-1-yl)ethanone **4** in ethanol with hydrazine hydrate gave a crystalline product that was identified as 5,6-dimethyl-1*H*-benzo[*d*]imidazole-2(3*H*)-thione **3** but 1-(2-hydrazono-5,6-dimethyl-2,3-dihydro-1*H*-benzo[*d*]imidazol-1-yl)ethanone did not form.

**Scheme 3:**
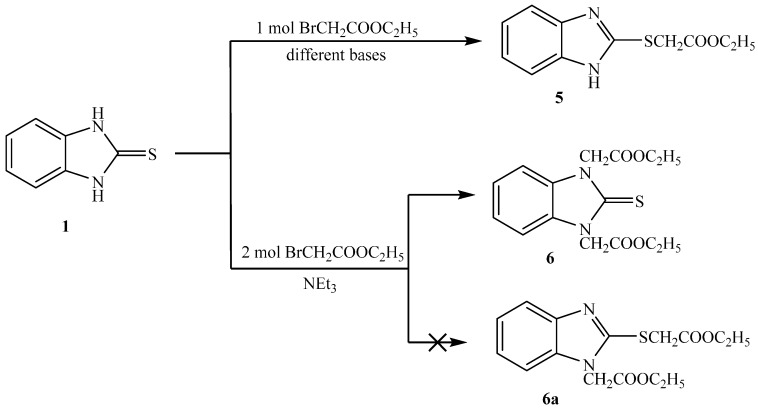
Synthesis of **5** and **6**.

Reaction of 1*H*-benzo[*d*]imidazole-2(3*H*)-thione **1** with ethyl bromoacetate, in presence of different bases in dry acetone gave ethyl 2-(1*H*-benzo[*d*]imidazol-2-ylthio)acetate **5** (Scheme 3). When using triethylamine as base, the product was obtained in high yield, but low yield was obtained when potassium carbonate was used [18]. The structure of the compound is consistent with the expected product. The IR data showed bands at ν 3457 (NH), 1739 (C=O), 1269, and 1167 cm^−1^ (C-O). Its ^1^H-NMR spectrum showed the presence of one NH as a singlet at δ 12.60 ppm, the methylene group show one singlet at δ 4.18 ppm connected with sulfur but not nitrogen. Its ^13^C-NMR spectrum showed the presence of one CH_3_ as a signal at δ 13.2, two CH_2_ as a signal at δ 32.0 and 60.4 ppm, and the presence of C=O at 167.8 ppm.

It was found that reaction of **1** with two equivalents of ethylbromoacetate, in the presence of triethylamine in dry acetone gave diethyl 2,2′-(2-thioxo-1*H*-benzo[*d*]imidazole-1,3(2*H*)-diyl)diacetate **6**. From spectral analysis, IR data showed bands at ν: 1739 (C=O) and 1055 cm^−1^ (C=S). Its ^1^H-NMR spectrum showed the presence of two methylene groups as one singlet at δ 5.16 ppm connected with two nitrogens and indicated the absence of NH signals. This confirmed that the two methylene groups were attached to the two nitrogen atoms but not attached to sulfur and nitrogen. Its ^13^C-NMR showed the presence of two CH_3_ as signal at δ 13.10, and two CH_2_ as signals at δ 44.00 and 60.40 ppm. The ^13^C-NMR showed the presence of C=S as signal at δ 166.20 and C=O as signal at δ 169.20 ppm

**Scheme 4:**
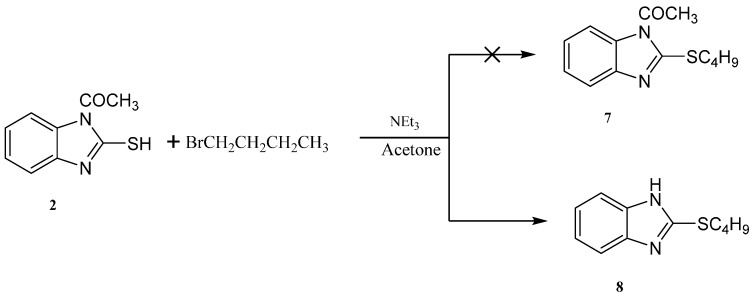
Synthesis of the 2-(butylthio)-1*H*-benzo[*d*]imidazole **8**.

**Figure 1:**
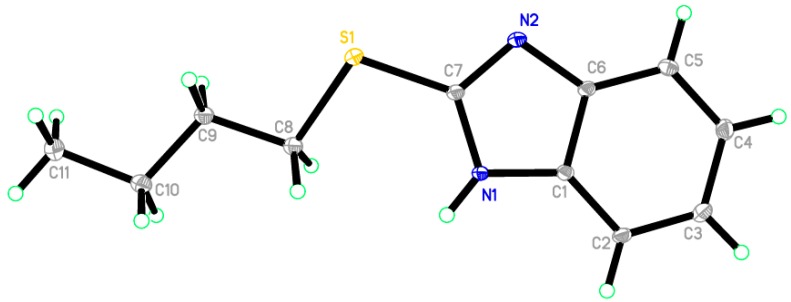
ORTEP diagram of the titled compound **8** drawn at 50% ellipsoids for non-hydrogen atom.

Reaction of 1-(2-mercapto-1*H*-benzo[*d*]imidazol-1-yl)ethanone **2** with 1-bromobutane in acetone as a solvent in the presence of triethylamine gave a product that was identified as 2-(butylthio)-1*H*-benzo[*d*]imidazole **8** (Scheme 4) [15]. Its IR spectrum showed the presence of a band at δ 3450 cm^−1^ (NH). Its ^1^H-NMR spectrum showed the presence of NH as a singlet at δ 12.47 ppm and did not show the presence of a methyl group. Its ^13^C-NMR spectrum showed the presence of CH_3_ at δ 12.5, and three sets of signals belong to CH_2_ at δ 20.3, 29.9 and 30.4 ppm. The compound 1-(2-(butylthio)-1*H*-benzo[*d*]imidazol-1-yl)ethanone **7** cannot be formed because the acetyl group was removed by base. The structure of **8** was confirmed by X-ray crystallography. The butyl group was found to exist in a zigzag conformation, as shown in Figure 1 which shows the Oak Ridge Thermal Ellipsoid Plot Program (ORTEP) for Crystal Structure.

**Scheme 5:**
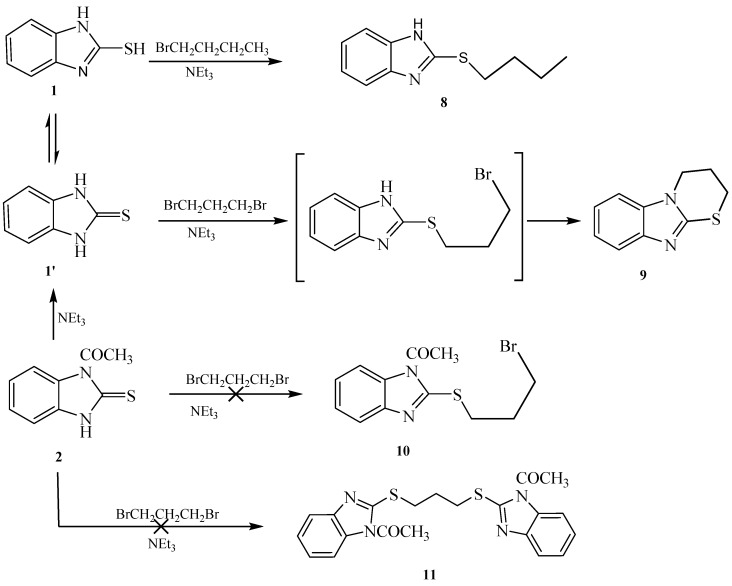
Synthesis of 3,4-dihydro-2*H*-[1,3]thiazino[3,2-*a*]benzimidazole **9**.

**Figure 2:**
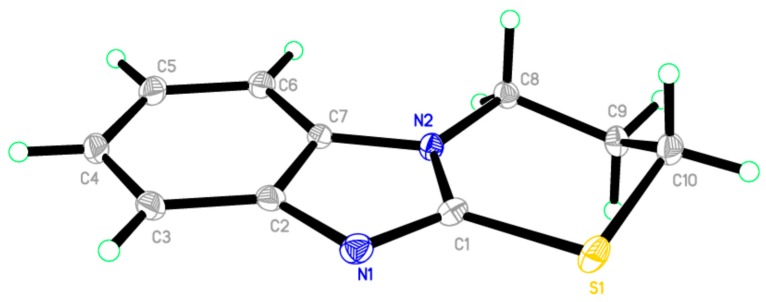
ORTEP diagram of the titled compound **9** drawn at 50% ellipsoids for non-hydrogen atoms.

Reaction of 1*H*-benzo[*d*]imidazole-2(3*H*)-thione **1** with dibromopropane, in ethanol as solvent and in the presence of triethylamine gave 3,4-dihydro-2*H*-[1,3]thiazino[3,2-*a*]benzimidazole **9** and not the 1,3-*bis*(1*H*-benzo[*d*]imidazol-2-ylthio)propane **10** [17] (Scheme 5). The ^1^H-NMR spectrum of the synthesized compound showed the presence of a methylene group at δ 3.25 ppm connected with sulfur, another methylene group at δ 4.18 ppm connected with nitrogen and the disappearance of signal belongs to NH. This confirmed that the alkylation occurred on the ring-sulfur that followed intramolecular cyclisation with the nitrogen atom of the same ring to give **9** and not with another ring to give **10**. The structure of **9** was confirmed by the X-ray crystallography (Figure 2). It showed that one of the CH_2_ is out of the plane of the ring system. Thus, the envelope conformation is the one having the minimized energy structure.

**Scheme 6:**
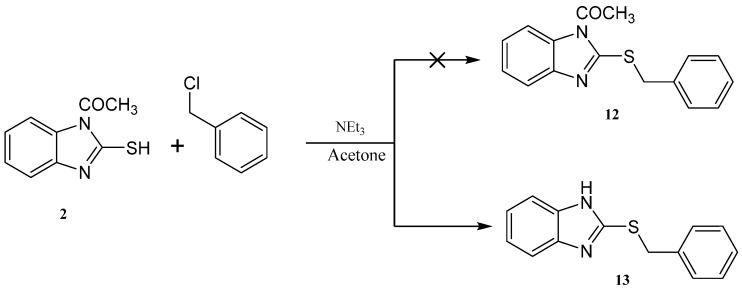
Synthesis of 2-(benzylthio)-1*H*-benzo[*d*]imidazole **13**.

Reaction of 1-(2-mercapto-1*H*-benzo[*d*]imidazol-1-yl)ethanone **2** with benzyl chloride in acetone as a solvent in the presence of triethylamine gave afford 2-(benzylthio)-1*H*-benzo[*d*]imidazole **13** (Scheme 6). The structure was confirmed from the spectral analysis [19]. Its IR spectrum showed the presence at 3400 cm^−1^ (NH), and did not show C=O band. The ^1^H-NMR spectrum showed the presence of NH as a singlet at δ 12.56 ppm, and the CH_2_ as a singlet at δ 4.50 ppm. Its ^13^C-NMR spectrum showed the presence of CH_2_ as a signal at δ 30.5, and the C=N at δ 149.1 ppm. The 1-(2-(benzylthio)-1*H*-benzo[*d*]imidazol-1-yl)ethanone **12** was not obtained because the acetyl group was removed by the base.

### 2.2. X-ray Single Crystal of ***8*** and ***9***

**Figure 3:**
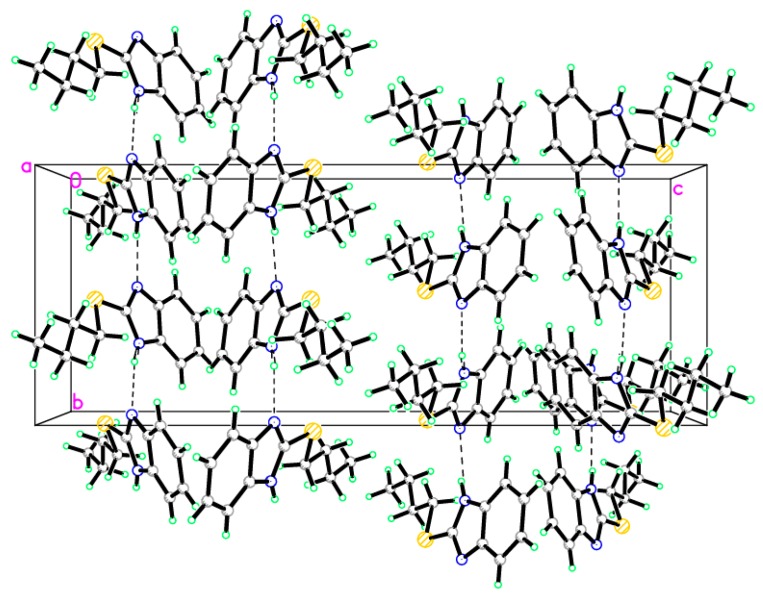
Crystal packing of compound **8** showing intermolecular hydrogen bonds as dashed lines along the *c*-axis.

**Figure 4:**
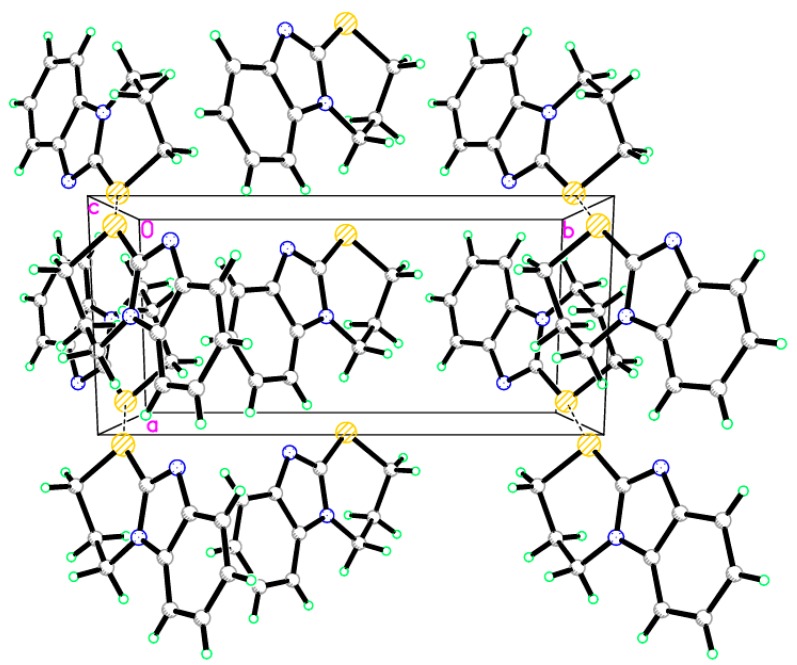
Crystal packing of compound **9**, dotted lines are short S···S interactions.

The structures of compounds **8** and **9** were unambiguously deduced by single-crystal X-ray diffraction (Figure 1, Figure 2, Figure 3 and Figure 4) technique. CCDC-1433060 and CCDC-1433059 contain the supplementary crystallographic data for this paper. These data can be obtained free of charge via http://www.ccdc.cam.ac.uk/conts/retrieving.html (or from the CCDC, 12 Union Road, Cambridge CB2 1EZ, UK; Fax: +44-1223-336033; E-mail: deposit@ccdc.cam.ac.uk. The structures were resolved by direct methods using the SHELXS97 program in the SHELXTL-plus package, and refined by a full-matrix least-squares procedure on *F*^2^ using SHELXS97 [20]. Diffraction data were collected on a Bruker SMART APEXII CCD diffractometer (Bruker AXS Advanced X-ray Solutions GmbH, Karlsruhe, Germany). The crystal structure and refinement data of compounds **8** and **9** are listed in Table 1. The selected bond distances and angles are presented in Table 2, Table 3 and Table 4. ORTEP drawings of final X-ray model of compounds **8** and **9** with the atomic numbering scheme are presented in Figure 1 and Figure 2, while crystal packing presentation of compounds **8** and **9** are shown in Figure 3 and Figure 4, respectively.

**Table 1:** The crystal and experimental data of compounds **8** and **9**.

Crystal Data	Compound 8	Compound 9
Empirical formula	C_11_H_14_N_2_S	C_10_H_10_N_2_S
Formula weight	206.30	190.27
Temperature	293 K	293 K
Wavelength	0.71073 Å	0.71073 Å
Crystal system	Orthorhombic	Monoclinic
Space group	*Pbca*	*P*2_1_/*c*
a	8.9060 (4)	6.0874 (3)
b	9.6531 (4)	12.3309 (6)
c	24.8720 (13)	12.4511 (6)
β	90.00	110.087 (3)
Volume	2138.26 (17)	877.77 (8)
Z	8	4
Calculated density	1.282 Mg·m^−3^	1.440 Mg·m^−3^
Absorption coefficient	0.26	0.32
F(000)	880	400
Crystal size	0.38 × 0.25 × 0.22 mm	0.68 × 0.53 × 0.40 mm
θ range	2.8° to 30.5°	2.4° to 30.6°
Reflections Collected	3278	2691
(*R*_int_)	0.087	0.066
*R*_1_ with I > 2σ (I)	0.056	0.035
*R*_2_ with I > 2σ (I)	0.142	0.091
Goodness of fit	1.20	1.06
max/min ρeA˚^−3^	0.77 and −0.29	0.42 and −0.33
CCDC number	1433060	1433059

**Table 2:** Selected geometric parameters (Å, °) of **8**.

Bond Length or Angle	(Å, °)	Bond Length or Angle	(Å, °)
S1—C7	1.7350 (18)	N1—C7	1.365 (2)
S1—C8	1.8070 (19)	N2—C6	1.393 (2)
N1—C1	1.385 (2)	N2—C7	1.325 (2)
C7—S1—C8	104.03 (9)	N2—C6—C1	109.87 (15)
C1—N1—C7	106.36 (15)	S1—C7—N2	120.67 (13)
C6—N2—C7	104.41 (15)	N1—C7—N2	113.81 (16)
N1—C1—C2	132.08 (16)	S1—C7—N1	125.46 (13)
N1—C1—C6	105.54 (15)	S1—C8—C9	105.48 (13)
N2—C6—C5	130.10 (16)		

**Table 3:** Hydrogen-bond geometry (Å, °) of **8**.

D—H···A	D—H	H···A	D···A	D—H···A
N1—H1N1···N2 ^i^	0.86 (3)	2.07 (3)	2.886 (2)	159 (3)
Symmetry code: (i) – x + 1/2, y + 1/2, z.				

**Table 4:** Selected geometric parameters (Å, °) of **9**.

Bond Length or Angle	(Å, °)	Bond Length or Angle	(Å, °)
S1—C1	1.7402 (11)	N2—C1	1.3700 (16)
S1—C10	1.8174 (12)	N2—C7	1.3850 (14)
N1—C1	1.3226 (15)	N2—C8	1.4654 (15)
N1—C2	1.3925 (15)		
C1—N1—C2	104.02 (10)	N1—C2—C3	130.19 (11)
C1—N2—C7	105.90 (9)	N1—C2—C7	110.22 (10)
C1—N2—C8	128.83 (9)	N2—C7—C2	105.67 (10)
C7—N2—C8	125.04 (10)	N2—C7—C6	131.10 (11)
S1—C1—N1	122.49 (9)	N2—C8—C9	111.91 (10)
S1—C1—N2	123.29 (8)	S1—C10—C9	111.49 (8)

## 3. Materials and Methods

### General Methods

The stated melting points are uncorrected and were performed on Gallenkamp melting point apparatus (Toledo, OH, USA). The purities of the compounds were checked by TLC using Merk Kieselgel 60-F254 plates (Darmstadt, Germany), and visually detected in an iodine chamber. The structures of the synthesized compounds were elucidated by using IR spectra on FT-IR (Shizmadu-series, Kyoto, Japan) using KBr disc technique at spectral laboratories in King Saud University. ^1^H-NMR spectra were determined with Jeol spectrometer (Tokyo, Japan) at 500 MHz and expressed in δ units (ppm) relative to an internal standard of tetramethylsilane in solvent DMSO-*d*_6_ at Alexandria University. ^13^C-NMR spectra were recorded with Jeol spectrometer at 125.7 MHz. The chemical shifts are expressed in δ (ppm) relative to the reference tetramethylsilane. The DMSO-*d*_6_ was used as a solvent. Elemental analyses were performed at the micro-analytical laboratory at Cairo University, Egypt.

#### 1-(2-Thioxo-2,3-dihydro-1*H*-benzo[*d*]imidazol-1-yl)ethanone **2**

Acetic anhydride (30 mL, 0.033 mol) was added to 1*H*-benzo[*d*]imidazole-2(3*H*)-thione **1** (5 g, 0.033 mol) and the stirred mixture was heated to 110–115 °C for 30 min. The solution was cooled then water (150 mL) was added, and finally kept for 30 min at room temperature. Colorless crystals were filtered off (6.21 g, 97% Yield); TLC, R_f_ = 0.773 (1:1, *n*-hexane:ethyl acetate). The product was recrystallized from benzene to give white crystals. m.p. 201–202 °C, (lit. [21] m.p., 195 °C); IR (KBr) ν 1716 (C=O), 1368 (CH_3_), 2996 (CH aliphatic), and 3146 (CH aromatic), 1591 cm^−1^ (C=C). ^1^H-NMR (CDCl_3_/D_2_O) δ: 1.92 (s, 3H, CH_3_), 7.19 (m, 2H, Ar-H), and 7.26 ppm (m, 2H, Ar-H). ^13^C-NMR (DMSO-*d*_6_) δ: 27.1 (CH_3_), 108.5 (CH), 114.3 (CH), 122.3 (CH), 124.3 (CH), 129.8 (C), 130.1 (C), 168.9 (C=S), and 171.1 ppm (C=O).

#### 1-(5,6-Dimethyl-2-thioxo-2,3-dihydro-1*H*-benzo[*d*]imidazol-1-yl)ethanone **4**

Acetic anhydride (0.002 mol, 4 mL) was added to (0.002 mol, 0356 g) of 5,6-dimethyl-1*H*-benzo[*d*]imidazole-2(3*H*)-thione **3** and the mixture was heated for 0.5 h. The solution was cooled, stirred and poured onto water, and finally kept for 30 min at room temperature. Colorless sparkling crystals were filtered off (0.39 g, 89% yield), TLC, R_f_ = 0.966 (1:1, *n*-hexane:ethyl acetate). The product was recrystallized from ethanol, m.p. 258–259 °C; IR (KBr) ν 1685 (N-C=O), 1627 (C=N), 1506 (C=C), 3192 cm^−1^ (C-H aromatic). ^1^H-NMR (DMSO-*d*_6_) δ: 2.22 (s, 6H, 2CH_3_), 2.94 (s, 3H, CH_3_), 6.88 (s, 1H, Ar-H), 7.76 (s, 1H, Ar-H), and 13.14 ppm (s, 1H, NH). ^13^C-NMR (DMSO-*d*_6_) δ: 18.6 (CH_3_), 18.9 (CH_3_), 27.1 (CH_3_), 109.0 (CH), 115.0 (CH), 128.2 (C), 130.7 (C), 132.9 (C), 168.4 (C=S), and 171.1 ppm (C=O). Calc. for C_11_H_12_N_2_OS (220.29); C, 59.97; H, 5.49; N, 12.72%, Found C, 59.66; H, 5.23; N, 12.43%

#### Ethyl 2-(1*H*-benzo[*d*]imidazol-2-ylthio)acetate **5**

A mixture of 1*H*-benzo[*d*]imidazole-2(3*H*)-thione **1** (0.01 mol, 1.5 g), in dry acetone 25 mL and potassium carbonate (0.01 mol, 1.62 g) was stirred and heated under reflux for 1 h. Ethyl bromoacetate (0.01 mol, 1.67 g, 1.1 mL) was added to the reaction mixture and continuing stirring and heating for another 15 h until completion of the reaction. The reaction mixture was cooled then filtered off. Water was added to the filtrate and left at room temperature for 24 h. The precipitate was filtered off and washed with water to give product, yield 77%. It was recrystallized from ethanol to give white crystal, m.p. 97–98 °C (Lit. [18] m.p. 60–62 °C, Lit. [15] m.p. 117 °C), TLC, R_f_ = 0.554 (1:1, *n*-hexane:ethyl acetate) IR (KBr) ν 3457 (NH), 1507 (NH) IP, 1739 (C=O), 1269, 1167 (C-O), 3150 (CH aromatic), and 1591 cm^−1^ (C=C). ^1^H-NMR (DMSO-*d*_6_) δ: 1.13 (t, 3H, CH_3_), 4.09 (q, 2H, CH_2_), 4.18 (s, 2H, CH_2_), 7.1 (s, 2H, Ar-H), and 7.40 (d, 2H, Ar-H), 12.60 ppm (s, 1H, NH). ^13^C-NMR (DMSO-*d*_6_) δ: 13.2 (CH_3_), 32.0 (CH_2_), 60.4 (CH_2_), 120.7 (Ar-C), 148.3 (C=N), and 167.8 ppm (C=O).

#### Diethyl 1,3-(2-thioxo-1*H*-benzo[*d*]imidazole-1,3(2*H*)-diyl)diacetate **6**

A mixture of 1*H*-benzo[*d*]imidazole-2(3*H*)-thione **1** (0.01 mol, 1.5 g), in dry acetone (25 mL) and triethylamine (0.01 mol, 1.7 mL) was stirred and heated under reflux for 1 h. Ethylbromoacetate (0.02 mol, 2.2 mL) was added to the reaction mixture and continuing stirring and heating for another 19 h until completion of the reaction. The reaction mixture was cooled then filtered off. Water was added to the filtrate and left at room temperature for 24 h till precipitate was filtered off with water. The product was white powder, yield 76%. It was recrystallized from ethanol. m.p. 199–201 °C, TLC, R_f_ = 0.722 (1:1, *n*-hexane:ethyl acetate), IR (KBr) ν 3457 (NH), 1507 (NH) IP, 1739 (C=O), 1220, 1093 (C-O), 1055 (C=S), 3058 (CH aromatic), and 1617 cm^−1^ (C=C); ^1^H-NMR (DMSO-*d*_6_) δ: 1.18 (t, 6H, 2CH_3_), 4.14 (q, 4H, 2CH_2_), 5.16 (s, 4H, 2CH_2_), 7.25 (m, 2H, Ar-H), and 7.47 ppm (m, 2H, Ar-H). ^13^C-NMR (DMSO-*d*_6_) δ: 13.1 (2CH_3_), 44 (2CH_2_), 60.4 (2CH_2_), 108.9 (2CH), 122.3 (2CH), 130.7 (2C), 166.2 (2C=O), and 169.2 ppm (C=S). Calc. for C_15_H_18_N_2_O_4_S (322.38): C, 55.88; H, 5.63; N, 8.69%. Found; C, 55.88; H, 5.63; N, 8.69%.

#### 2-(Butylthio)-1*H*-benzo[*d*]imidazole **8**

To a stirred solution of 1-bromobutane (0.005 mol, 0.54 mL) in 10 mL of acetone was added a solution of 1-(2-mercapto-1*H*-benzo[*d*]imidazol-1-yl)ethanone **2** (0.005 mol, 0.96 g) in 40 mL acetone containing triethylamine (0.01 mol, 1.4 mL). The reaction mixture was stirred for 28 h at room temperature, then evaporated under vacuum, water was added to the precipitate, filtered off and the product (0.64 g, 52% yield); TLC, R_f_ = 0.601 (1;1, *n*-hexane:ethyl acetate) and was crystallized from ethanol to give 2-(butylthio)-1*H*-benzo[*d*]imidazole **8**, m.p. 135 °C (135 °C lit. [19], 134–135 °C lit. [22]); IR (KBr) ν 3400 (NH), 1671 (C=N), 1619 (NH-IP), 1588 (C=C), 3047 (CH-aromatic), 2955 (CH aliphatic), and 1498, 1465, 1432.7 cm^−1^ (CH_2_). ^1^H-NMR (DMSO-*d*_6_) δ: 0.85 (t, 3H, CH_3_), 1.37 (sextet, 2H, CH_2_), 1.63 (quint, 2H, CH_2_), 3.23 (t, 2H, CH_2_), 7.06 (m, 2H, Ar-H), 7.50 (s, 2H, Ar-H), and 12.50 ppm (s, IH, NH). ^13^C-NMR (DMSO-*d*_6_) δ: 12.52 (CH_3_), 20.3 (CH_2_), 29.9 (CH_2_), 30.4 (CH_2_), 120.4 (Ar), and 149.3 ppm (C=N). Calc. for: C_11_H_14_N_2_S (206.31); C, 64.04; H, 6.84; N, 13.58%, Found C, 64.32; H, 6.34; N, 13.26%.

#### 3,4-Dihydro-2*H*-[1,3]thiazino[3,2-*a*]benzimidazole **9**

A mixture of 1*H*-benzo[*d*]imidazole-2(3*H*)-thione **1** (0.026 mole, 5 gm) in 60 mL ethanol and in presence of triethylamine (0.01 mol, 1.39 mL) was refluxed for 1 h, then 1,3-dibromopropane (0.013 mol, 2.6 g) was added. The reaction mixture was further heated under reflux for 5 h. Then, ethanol was removed under vacuum, and water (20 mL) was added to the product, and kept for 24 h at room temperature to give white crystals. The product (4.68 g, 83% yield), was recrystallized from ethanol, TLC, R_f_ = 0.382 (1:1, *n*-hexane:ethyl acetate), m.p. 201–202 °C. ^1^H-NMR (DMSO-*d*_6_) δ: 2.29 (m, 2H, CH_2_), 3.26 (t, 2H, CH_2_), 4.17 (t, 2H, CH_2_), 7.11 (m, 2H, Ar-H), and 7.39 ppm (m, 2H, Ar-H). ^13^C-NMR (DMSO-*d*_6_) δ: 21.9 (CH_2_), 24.3 (CH_2_), 41.7 (CH_2_), 107.9 (CH), 116.1 (CH), 120.02 (CH), 120.94 (CH), 134.69 (C), 134.81 (C), 141.4; 145.75 ppm (C=N). Calc. for C_10_H_10_N_2_S (190.26): C, 63.13; H, 5.30; N, 14.72%. Found: C, 63.42; H, 5.11; N, 14.41%.

#### 2-(Benzylthio)-1*H*-benzo[*d*]imidazole **13**

To a stirred solution of benzyl chloride (0.005 mol, 0.57 mL) in 10 mL of acetone was added a solution of 1-(2-mercapto-1*H*-benzo[*d*]imidazol-1-yl)ethanone (0.005 mol, 0.96 g) in 40 mL acetone containing triethylamine (0.01 mol, 1.39 mL). The reaction mixture was stirred for 30 h at room temperature, then evaporated under vacuum, water was added to the precipitate, filtered off and the product (0.4 g, 74% yield), TLC, R_f_ = 0.644 (1:1, *n*-hexane: ethyl acetate) was recrystallized from ethanol to give 2-(benzylthio)-1*H*-benzo[*d*]imidazole, m.p. 185–186 °C (lit. [19] m.p. 184 °C, lit. [23]. m.p. 184–185 °C); IR (KBr) ν 3400 (NH), 1585 (NH-IP), 1453 (CH_2_), 2963 (CH aliphatic), 3070 (CH aromatic), and 1610, 1515 cm^−1^ (C=C). ^1^H-NMR (DMSO-*d*_6_); δ: 4.50 (s, 2H, CH_2_), 7.08 (m, 2H. Ar-H), 7.21 (t, 1H, Ar-H), 7.30 (t, 2H, Ar-H), and 7.41–7.51 ppm (bd, 4H, Ar-H). ^13^C-NMR (DMSO-*d*_6_); δ: 30.5 (CH_2_), 120.8 (CH), 126.7 (CH), 127.9 (CH), 128.2 (CH), 137.0 (C), and 149.1 ppm (C=N).

## 4. Conclusions

The benzimidazole ring has been considered a pharmacophore ring. It has active centers that can be modified by some chemical reactions such as alkylation and acetylation to provide compounds of potential biological activity. The alkylation of 1*H*-benzo[*d*]imidazole-2(3*H*)-thione with dibromopropane did not give the respective alkylated derivatives 2-(3-bromopropylthio)-1*H*-benzo[*d*]imidazole or 1,3-*bis*(1*H*-benzo[*d*]imidazol-2-ylthio)propane, but gave 3,4-dihydro-2*H*-[1,3]thiazino[3,2-*a*]benzimidazole. The formation of the last tricyclic ring indicated that the alkylation that occurred on the ring-sulfur atom was followed by intramolecular cyclisation with the nitrogen atom of the benzimidazole ring. This was confirmed by the X-ray crystallography. Alkylation of benzimidazole thione and its acetyl derivative gave the corresponding *S*-alkylated products, and in some cases a deacetylation process has been occurred. In the case of using ethyl bromacetate, it gave *S*-mono- or *S*-, *N*-dialkylated derivatives depending on the ratio of the molar equivalents of the reactants. Deacetylation of acetyl benzimidazolethione derivative by different bases gave the imidazole thione, together with the starting acetyl-benzimidazolethione. However, increasing time caused a gradual change in their ratio till complete conversion to the deacetylated derivative. Both 1*H*-benzo[*d*]imidazole-2(3*H*)-thione and 5,6-dimethyl-1*H*-benzo[*d*]imidazole-2(3*H*)-thione can exhibit tautomerism, and it has also been proven that the compounds in the solid state and in solution exist in the thione form, as confirmed by ^1^H-NMR spectra.
